# Luteotropic and Luteolytic Factors Modulate the Expression of Nuclear Receptor Coregulators in Bovine Luteal Cells Independently of Histone Acetyltransferase and Histone Deacetylase Activities

**DOI:** 10.3390/ani13172784

**Published:** 2023-08-31

**Authors:** Robert Rekawiecki, Michal Hubert Wrobel, Paulina Zajac, Oliwia Serej, Magdalena Karolina Kowalik

**Affiliations:** Institute of Animal Reproduction and Food Research of the Polish Academy of Sciences, Tuwima 10, 10-747 Olsztyn, Poland; m.wrobel@pan.olsztyn.pl (M.H.W.); p.zajac@pan.olsztyn.pl (P.Z.); o.serej@pan.olsztyn.pl (O.S.); m.kowalik@pan.olsztyn.pl (M.K.K.)

**Keywords:** progesterone receptor coregulators, P300, CREB, SRC-1, NCOR-2, corpus luteum, cow

## Abstract

**Simple Summary:**

Proper functioning of the ovaries significantly affects the duration of the estrous cycle and the appropriate course of pregnancy in female livestock, including cattle. The determinant of these processes is progesterone (P4), which, on the genomic pathway, acts through nuclear receptors that, by attaching to the promoter of a target gene, activate its transcription. At the final stage of the activation of PGRs and other nuclear receptors are attached coregulators, elements that regulate receptor function. They are divided into coactivators and corepressors, and their attachment determines the activation or inhibition of transcription of the target gene activated by these receptors, respectively. Adequate levels and activities of coregulators are crucial for the function of nuclear receptors, including PGRs, and as a result, the course of the normal estrous cycle and maintenance of early pregnancy. Therefore, the results obtained may be helpful in better understanding the regulation and expression of coactivators and corepressors in the CL of cows and may contribute to reducing nuclear receptor dysfunction in PGRs. This may also have important practical significance, contributing to increasing the efficiency of breeding these animals by reducing the early embryonic lethality that results from abnormal functioning of the PGRs.

**Abstract:**

The aims of this study were to examine the effect of luteotropic and luteolytic factors on the mRNA and protein expression of the coactivators HAT: histone acetyltransferase p300 (P300), cyclic adenosine monophosphate response element-binding protein (CREB), and steroid receptor coactivator-1 (SRC-1) and the corepressor: nuclear receptor corepressor-2 (NCOR-2) in bovine luteal cells on days 6–10 and 16–20. HAT and HDAC activities were also measured. The obtained results showed that luteotropic and luteolytic factors influence changes in the mRNA and protein levels of the coregulators of PGRs. However, they did not affect the activity of related HAT and HDAC, respectively. Therefore, it is possible that these factors, through changes in the expression of nuclear receptor coactivators and corepressors, may affect the functioning of the nuclear receptors, including PGRs, in the bovine CL.

## 1. Introduction

The corpus luteum (CL) is an endocrine structure that is the main producer of progesterone (P4), a hormone responsible for regulating the estrous cycle duration and maintaining pregnancy. It uses two mechanisms of action in these processes: genomic, through nuclear receptors, and nongenomic, through membrane receptors [1]. Nuclear P4 receptors (PGRs) exist in two main isoforms transcribed from the same gene but with different promoters, referred to as isoform A (PGRA) and B (PGRB). PGRB is the longer isoform and varies by over a hundred amino acids across species. The relative expression of *PGRA* and *PGRB* varies by species. It was demonstrated that mRNA levels of *PGRB* in cows were 500–2000 times lower than RNA levels for the *PGRA* isoform [2]. Another difference between isoforms is their mode of action. The primary P4 action occurs through PGRB, while PGRA acts to inhibit the action of PGRB. The final step in PGR activation occurs in the cytoplasm and involves the attachment of proteins called coregulators to the receptor. This group of proteins includes coactivators, whose binding to a PGR results in its activation, as well as corepressors, whose binding to a PGR has the opposite effect [3,4]. The action of coregulators is not limited only to PGRs. They can also regulate the activity of other steroid hormone receptors, such as those for estradiol (E2) [5], androgen [6], and glucocorticoid [7]. The coactivators show histone acetyltransferase (HAT) activity, attaching acetyl groups onto the lysine residues of histone proteins and leading to chromatin relaxation [8]. In contrast, corepressors show histone deacetylase (HDAC) activity, deacetylating lysines in histone proteins and leading to chromatin packing [9].

We have previously shown that coactivators and corepressors demonstrate variable expression during the estrous cycle in the CL [10] and endometrium [11]. We have also presented that luteotropic factors (luteinizing hormone (LH), prostaglandin E2 (PGE2), and E2) and luteolytic factors (prostaglandin F2α (PGF2α) and nitric oxide) regulate PGRA and PGRB expression [12]. The existence of the CL depends on the state of imbalance between the influence of luteotropic factors, which support CL function, and luteolytic factors including PGF2α produced in the uterus. The predominance of luteotropic factors causes the produced P4 to create appropriate conditions for implantation and development of the embryo in the uterus and provides atony of the uterus by blocking the synthesis of oxytocin receptors (ROT). One of the most important luteotropic regulators in the cows’ CL is LH. An increase in the level of this hormone leads to the rupture of the ovarian follicle and is responsible for the luteinization of the corpus luteum and an increase in P4 synthesis [13]. Another of the luteotropic factors that regulate CL function is PGE2. The highest production of this prostaglandin was detected in the early luteal phase, so this indicates its protective effect on the CL against luteolysis, and it may also influence P4 secretion by the CL [14]. The opposite effect is shown by PGF2α, which results in functional and morphological regression of the CL. During this time, it is also important to reduce the effect of luteotropic factors on the CL, which activates the secretion of the uterine PGF2α and transports it to the CL [15]. Luteal regression is also characterized by an increased peripheral concentration of estradiol (E2), which leads to the initiation of the preovulatory LH surge in cattle CL [16]. Additionally, the promoter of the *PGR* gene includes a sequence referred to as the estrogen response element (ERE), which binds E2 [17]. This indicates that the hormone is involved in PGR activation. The main hormone affecting CL function is P4. This hormone increased the secretion of PGE2, and this in turn caused an increase in P4 levels on days 5–10 in the cow’s CL [14]. It also influenced its own synthesis in the CL by increasing 3β-hydroxysteroid dehydrogenase (3β-HSD) activity on days 5–10 of the estrous cycle during intensive CL growth. P4 has also been shown to influence its own synthesis at the mRNA level by stimulating the CL steroidogenic acute regulatory protein (*StAR*), cytochrome P450scc (*P450scc*), and *3β-HSD* gene expression on days 6–16 of the estrous cycle [18]. The action of P4 is blocked by Onapristone (ZK299) and Mifepristone (RU486), which differently interact with the PGR. ZK299 binds to a PGR and prevents its interaction with the target gene’s promoter [19]. In contrast, RU486 binds to a PGR and interferes with its activity [20]. Nitric oxide donor (2S)-2-pentylbutanedioic acid (NONate) is a compound that regulates blood flow and stimulates PGF2α synthesis in a cow’s CL [21]. It also exerts anti-luteolytic properties by inhibiting the 9-keto-PGE2 reductase, which converts PGE2 to PGF2a, as demonstrated in sheep endometrium [22]. Aminoglutethimide (AMG) inhibits enzymes such as the cholesterol side-chain cleavage enzyme (CYP11A1, P450scc) and aromatase (CYP19A1), thus inhibiting the entire process of steroidogenesis [23]. Thus, a shift in the predominance of action toward luteolytic or luteotropic factors involves a change in CL function. This process may also affect the mRNA and protein levels of coregulators and corepressors and modulate HAT and HDAC activities. These changes may affect PGR action and in consequence the effect of P4 on luteal cells. Therefore, this study aimed to determine the effect of LH, PGE2, E2, P4, PGF2α, RU486, ZK299, NONate, and AMG on mRNA and protein levels of the nuclear receptor coactivators: histone acetyltransferase p300 (P300), cyclic adenosine monophosphate response element-binding protein (CREB), and steroid receptor coactivator-1 (SRC-1) and the nuclear receptor corepressor: nuclear receptor corepressor-2 (NCOR-2) in bovine luteal cells on days 6–10 and 17–20. It further aimed to determine the HAT and HDAC activity in luteal cells under the influence of these factors and the concentrations of HAT and HDAC inhibitors for use in future studies.

## 2. Materials and Methods

### 2.1. CL Collection and Isolation of Luteal Cells

CLs from days 6 to 10 (mid-luteal, with high P4 level) and 17 to 20 (luteal regression, with low P4 level) of the estrous cycle were selected based on our previous research [10]. The days of the estrous cycle were assessed based on morphological observations of the ovaries (color, blood supply, CL size and diameter, and size of ovarian follicles) and the uterus (color and plumpness of the endometrium; amount, color, consistency, and clarity of mucus inside the uterine cavity; and size and color of the endometrial glands) based on criteria given by Ireland [24,25]. Corpora lutea samples from days 6 to 10 were characterized by apex red or brown, while the rest of the CL was orange. There was visible surface vascularization and they measured 10–15 mm diameter. From days 17 to 20, CLs were characterized by a surface in color from orange to yellow. Vasculature on the surface of CLs was not visible and the diameter of CLs was approximately 1 cm. They were obtained from non-pregnant meat cows from a local slaughterhouse and transported to the laboratory in ice-cold saline within 1 h after slaughter. Single luteal cell isolation involved perfusion and pooling of 3 CLs immediately after the material was delivered to the laboratory. CLs were perfused with a mixture of collagenase IA (1 mg/mL) and DNase I (5 µg/mL) in Medium 199 with a temperature of 37.5 °C, as described by [26]. Perfusion with the collagenase solution was carried out for about 40 min until a mushy structure was obtained. Then, the CL tissue was dispersed with a scalpel and digested in collagenase solution for a further 30 min. The obtained cell mixture was washed three times by centrifugation with Medium 199. Trypan blue (0.04%) dye exclusion assay was used to determine luteal cell viability. Cells showing >85% viability were used for further experiments. Next, cells in Dulbecco’s Modified Eagle Medium (DMEM)/Ham’s-F12 medium supplemented with 10% fetal calf serum were seeded into 6-well plates (Thermo Fisher Scientific; Wilmington, DE, USA) at a number of 10^6^ cells in 4 mL per one well of the plate. Cells were pre-incubated for 24 h in an atmosphere containing 5% CO_2_ and 100% humidity at a temperature of 38 °C (Heraeus GmbH, Hanau, Germany) to enable attachment of cells to the plate. Then, the cells were washed, and the medium was replaced with DMEM/Ham’s-F12 supplemented with 0.1% bovine serum albumin, ascorbic acid (20 μg/mL), transferrin (5 μg/mL), and sodium selenite (5 ng/mL; ICN, Poland). Luteal cells used in each experiment were pooled from 2–3 CLs.

Cells were stimulated at times selected on the basis of previous research [18] for 6 h for genes and 24 h for protein expression determination with the luteotropic factors: LH (100 ng/mL), progesterone (P4) (10^−5^ M), prostaglandin E2 (PGE2) (10^−6^ M), and estradiol (E2) (10^−8^ M) and the luteolytic factors: prostaglandin F2α (PGF2α) (10^−6^ M), progesterone receptor inhibitors: Mifepristone (RU486) (10^−5^ M) and Onapristone (ZK299) (10^−5^ M) as well as nitric oxide donor (NONate) (10^−4^ M), steroidogenesis inhibitor: aminoglutethimide (AMG 1.5 × 10^−4^ M) and selected HAT and HDAC inhibitors at the concentrations determined in the preliminary experiment. All experiments were carried out in groups with *n* = 4 and each treatment was tested in duplicate on the cell culture plate. Concentrations of LH and PGE2 were established in our previous research [18,27]. Doses of progesterone receptor inhibitors were used in studies on the function of PGR isoforms [28]. The other concentrations that were used in these studies had a modulating effect on the level of P4 (NONate and AMG [29], PGF2α [30], and E2 [31]).

### 2.2. P4 Concentrations

The enzyme immunoassay (EIA) method was used to determine P4 levels in the medium using absorbance at 450 nm in a microplate reader (Multiscan EX; Labsystem; Helsinki, Finland). P4 labeled with horseradish peroxidase (HRP) was applied in a dilution of 1:40,000, and its recovery averaged 90%. P4 antiserum was used in a dilution of 1:60,000, as was shown by [32]. The standard curve range was 0.1–25 ng/mL, while the method’s sensitivity was 0.15 ng/mL. The control samples had intra- and inter-assay coefficients of variation of 6.8% and 7.1%, respectively. The correlation between the quantity of P4 inserted and quantified for 4 different concentrations was significant (r = 0.96; *p* < 0.001).

### 2.3. RNA Isolation and Reverse Transcription

Total RNA was isolated using the Universal RNA Purification Kit (EURx, Gdansk, Poland) according to the manufacturer’s instructions. The concentration and quality of RNA were determined using a NanoDrop 1000 spectrophotometer (Thermo Fisher Scientific, Waltham, MA, USA) before being stored at −80 °C until required. RNA (1 µg) was subjected to a DNAase reaction to degrade the remaining DNA. Complementary DNA (cDNA) was synthesized via reverse transcription with the TRANSCRIPTME cDNA Synthesis Kit (Blirt, Gdansk, Poland) according to the manufacturer’s protocols.

### 2.4. Real-Time Polymerase Chain Reactions (PCRs)

The 7900 Real-Time PCR System (Applied Biosystems; Foster City, CA, USA) was used to carry out real-time PCR studies. The reactions were made with the Power SYBR Green PCR Master Mix (Applied Biosystems). Oligonucleotide PCR primers and expected amplicon lengths for coregulators *P300, CREB*, *SRC-1*, and *NCOR-2*, with the *TATA box-binding protein* (*TBP*) used as a housekeeping gene, are listed in Table 1. The reaction mixture (20 µL) consisted of 100 ng cDNA, 10 μL Master Mix (Applied Biosystems), and 0.2 mM for each gene-specific primer. The PCR reaction started with a preliminary denaturation step (10 min at 95 °C) followed by 40 cycles of denaturation (15 s at 95 °C) and annealing/extension (1 min at 60 °C). All PCR reactions (*n* = 5) were performed in duplicate.

### 2.5. Western Blot Analysis

Protein extracts were obtained through homogenization of luteal tissues in the radioimmunoprecipitation assay (RIPA) buffer containing protease inhibitors (25 mM Tris hydrochloride (pH 7.6), 150 mM sodium chloride (NaCl), 1% Triton X-100, 1% sodium deoxycholate, and 0.1% sodium dodecyl sulfate (SDS)). Next, 100 mg of protein per sample/well was electrophoresed on a 10% SDS-polyacrylamide gel (SDS-PAGE) precast Stain-Free gel (Bio-Rad; Hercules, CA, USA). Then, proteins were wet-transferred from the gel onto an Immobilon polyvinylidene fluoride (PVDF) membrane (Millipore; Billerica, MA, USA). Next, PVDF was blocked using 5% non-fat dry milk in Tris-buffered saline containing Tween 20 (TBST) buffer (100 mM Tris hydrochloride, 0.9% NaCl, and 0.05% Tween 20). Then, the PVDF membranes were treated overnight at 4 °C with the primary rabbit antibodies anti-P300 (1:400; molecular weight of ~264 kDa; Cohesion Biosciences, London, UK), anti-CREB (1:130; molecular weight of ~265 kDa; Cohesion Biosciences, London, UK), anti-SRC-1 (1:200; molecular weight of ~152 kDa; Sigma-Aldrich, Poznan, Poland), and anti-NCOR-2 (1:400; molecular weight of ~274 kDa; OriGene Technology; Rockville, MD, USA). After incubation, the PVDF membranes were rinsed three times for 10 min with TBST buffer. Next, the PVDF membranes were subjected to incubation with a secondary HRP-conjugated anti-rabbit antibody (1:50,000; Sigma-Aldrich; Poznan, Poland). Finally, the PVDF membrane was visualized using the Clarity Western ECL Blotting Chromogenic Substrate (Bio-Rad). The proteolytic reaction creates light proportional to the amount of HRP-labeled antibodies present. The signal was detected with a ChemiDoc Imaging System (Bio-Rad), and the level of any of the tested proteins was normalized to the total protein loading of the level of protein in each well of the gel using the ImageLab 6.1 software (Bio-Rad). The stain-free method does not need the use of additional housekeeping protein antibodies [33].

### 2.6. HAT and HDAC Activities

Preliminary experiment. Based on previously published studies, different HAT and HDAC inhibitors were used at three concentrations to determine the optimal concentration. Luteal cells on days 6–10 were incubated for 24 h with the following HAT inhibitors and concentrations: garcinol [34] at 500, 250, and 125 μM; anacardic acid (AnAc) [35] at 750, 500, and 250 μM; and C646 [36] at 500, 250, and 125 μM. They were also incubated with the following HDAC inhibitors and concentrations: suberoylanilide hydroxamic acid (SAHA) [37] at 25, 12.5, and 6.25 μM; apicidin [38] at 100, 50, and 25 μM; and CAY10433 [39] at 500, 250, and 125 μM. Cell viability was checked using the Alamar Blue reduction test. Staurosporine (ST, 500 ng/mL) was used as a control reduction of AlamarBlue. Cells in which inhibitors did not reduce Alamar Blue were tested using a HAT Activity Colorimetric Assay Kit and HDAC Activity Colorimetric Assay Kit (BioVision; Milpitas, CA, USA) according to the manufacturer’s recommendations. We determined that 500 μM AnAc and 25 μM SAHA inhibitors were the most effective in inhibiting the activity of HAT and HDAC, respectively.

Main experiment. Luteal cells representing days 6–10 and 17–20 were isolated and seeded in 6-well plates. Cells were stimulated for 24 h with luteotropic factors (LH (100 ng/mL), P4 (10^−5^ M), PGE2 (10^−6^ M), and E2 (10^−8^ M)), luteolytic factors (PGF2α (10^−6^ M), RU486 (10^−5^ M), ZK299 (10^−5^ M), nitric oxide donor NONate (10^−4^ M), and AMG (1.5 × 10^−4^ M)), and selected HAT and HDAC inhibitors at the concentrations determined in the preliminary experiment. After stimulation, P4’s level in the medium was measured by EIA. HAT and HDAC activity were estimated using colorimetric assay kits.

HAT and HDAC enzyme activities were determined using the HAT Activity Colorimetric Assay Kit (Biovision; Milpitas, CA, USA) and HDAC Activity Colorimetric Kit (Biovision; Milpitas, CA, USA) according to the manufacturer’s protocols. The isolated nuclear protein extracts prepared above were used as the research material.

### 2.7. Data Analysis

Experimental data showing hormone concentrations, real-time PCR, and Western blots are presented on the graphs as the mean ± standard error of the mean (SEM). A one-way ANOVA analysis of variance was used for the statistical comparisons, using Tukey’s test. Real-time quantitative data from the experiments were analyzed using the real-time PCR Miner algorithm [40] and then normalized to *TBP*. Western blot measurements were normalized to the total amount of proteins separated on the SDS-PAGE gels using stain-free technology and protein chemiluminescent detection. The GraphPad Prism v.8.0 software (GraphPad Software, Inc.; San Diego, CA, USA) was used for all statistical analyses.

## 3. Results

### 3.1. P4 Concentrations

**Figure 1:**
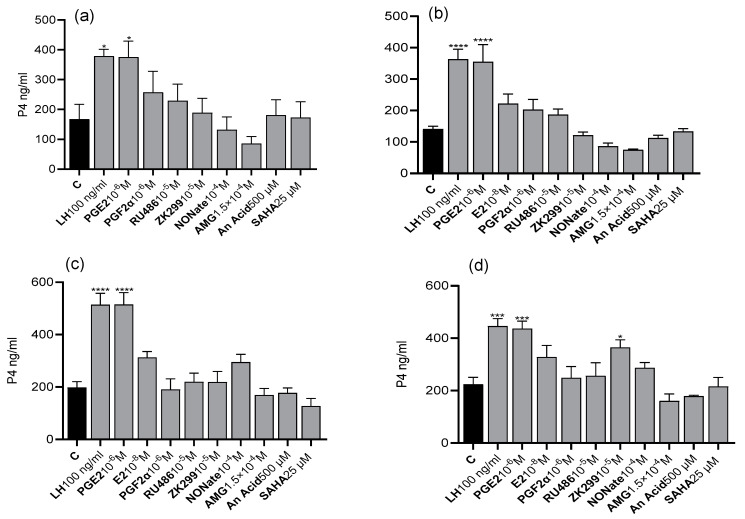
P4 concentration in luteal cells on days 6–10 (**a**,**c**) and 17–20 (**b**,**d**) after 6 h (**a**,**b**) and 24 h (**c**,**d**) of treatment with luteotropic factors: LH (100 ng/mL), prostaglandin E2 (PGE2) (10^−6^ M), and estradiol (E2) (10^−8^ M) and the luteolytic factors: prostaglandin F2α (PGF2α) (10^−6^ M), progesterone receptor inhibitors: Mifepristone (RU486) (10^−5^ M) and Onapristone (ZK299) (10^−5^ M) as well as nitric oxide donor (NONate) (10^−4^ M), steroidogenesis inhibitor: aminoglutethimide (AMG 1.5 × 10^−4^ M) and respectively selected HAT and HDAC inhibitors 500 μM anacardic acid (AnAc), and 25 μM suberoylanilide hydroxamic acid (SAHA) compared to untreated cells (C) (*n* = 4 in each group). Bars marked with asterisks are significantly different from the control (*, ***, and **** represent *p* < 0.05, *p* < 0.001, and *p* < 0.0001, respectively).

P4 levels in the medium increased after 6 h of incubation with LH (*p* < 0.05–0.0001) and PGE2 (*p* < 0.05–0.0001) for luteal cells on days 6–10 (Figure 1a) and on days 17–20 (Figure 1b). P4 levels in the medium of luteal cells from days 6 to 10 increased after 24 h of incubation with LH (*p* < 0.0001) and PGE2 (*p* < 0.0001) (Figure 1c). Similarly, P4 levels in the medium of luteal cells from days 17 to 20 increased after 24 h of incubation with LH (*p* < 0.001), PGE2 (*p* < 0.001), and ZK299 (*p* < 0.05) (Figure 1d).

### 3.2. Coregulator mRNA Levels

**Figure 2:**
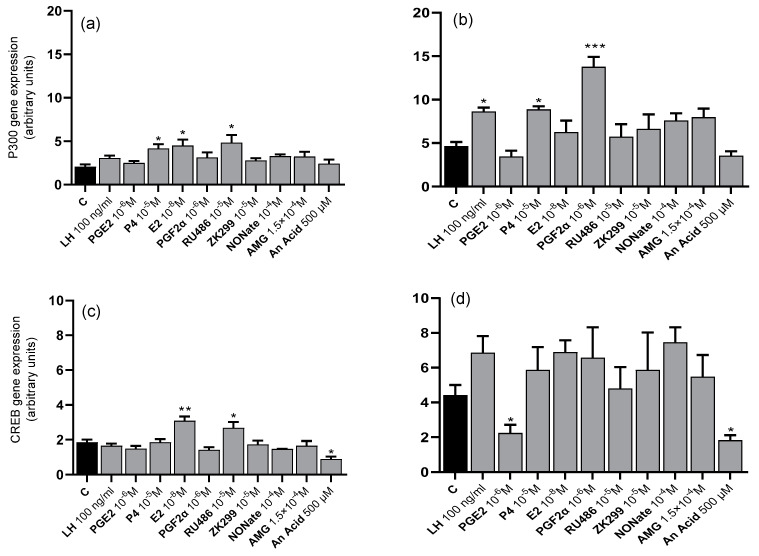

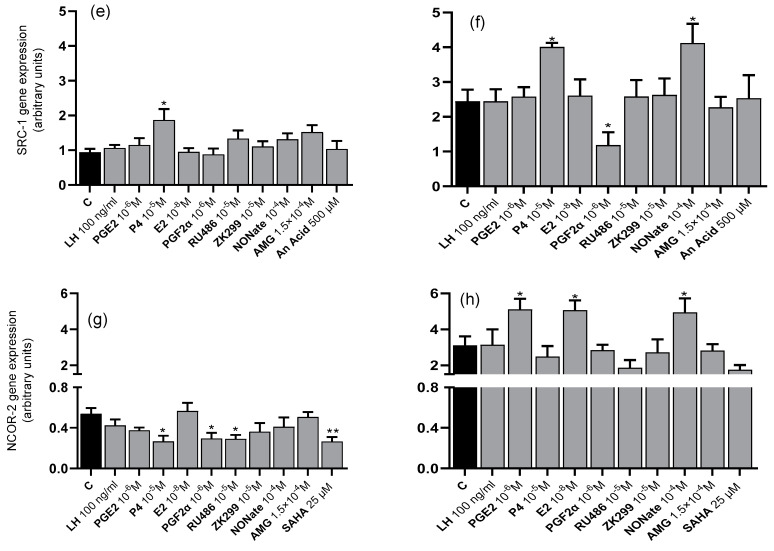
The mean (±SEM) of coactivators *P300* (**a**,**b**), *CREB* (**c**,**d**), and *SRC-1* (**e**,**f**) and corepressor *NCOR-2* (**g**,**h**) mRNA levels in luteal cells from days 6 to 10 (**a**,**c**,**e**,**g**) and 17 to 20 (**b**,**d**,**f**,**h**) after 6 h treatment with treatment with luteotropic factors:: LH (100 ng/mL); progesterone (P4) (10^−5^ M); prostaglandin E2 (PGE2) (10^−6^ M); estradiol (E2) (10^−8^ M) and the luteolytic factors: prostaglandin F2α (PGF2α) (10^−6^ M), progesterone receptor inhibitors: Mifepristone (RU486) (10^−5^ M) and Onapristone (ZK299) (10^−5^ M) as well as nitric oxide donor (NONate) (10^−4^ M); steroidogenesis inhibitor: aminoglutethimide (AMG 1.5 × 10^−4^ M) and respectively selected HAT and HDAC inhibitors 500 μM anacardic acid (AnAc), and 25 μM suberoylanilide hydroxamic acid (SAHA) compared to untreated cells (C) (*n* = 4 in each group). Bars marked with asterisks are significantly different from the control (*, **, and *** represent *p* < 0.05, *p* < 0.01, and *p* < 0.001, respectively).

*P300* coactivator mRNA levels in luteal cells from days 6 to 10 were increased by P4 (*p* < 0.05), E2 (*p* < 0.05), and RU486 (*p* < 0.05; Figure 2a), while in cells from days 17 to 20, *P300* mRNA levels were decreased by LH (*p* < 0.05), P4 (*p* < 0.05), and PGF2α (*p* < 0.001; Figure 2b). In luteal cells from days 6 to 10, *CREB* mRNA levels were increased by E2 (*p* < 0.01) and RU486 (*p* < 0.05) but decreased by the HAT inhibitor AnAc (*p* < 0.05; Figure 2c), whereas in cells from days 17 to 20, *CREB* mRNA levels were decreased by PGE2 (*p* < 0.05) and AnAc (*p* < 0.05; Figure 2d). In addition, *SRC-1* coactivator mRNA levels were increased by P4 (*p* < 0.05) in luteal cells from days 6 to 10 (Figure 2e) and by P4 (*p* < 0.05) and NONate (*p* < 0.05) in luteal cells from days 17 to 20 but decreased by PGF2α (*p* < 0.05; Figure 2f). Furthermore, *NCOR-2* corepressor mRNA levels were decreased in luteal cells from days 6 to 10 by P4 (*p* < 0.05), PGF2α (*p* < 0.05), RU486 (*p* < 0.05), and the HDAC inhibitor SAHA (*p* < 0.001; Figure 2g). However, they were increased in luteal cells from days 17 to 20 by PGE2 (*p* < 0.05), E2 (*p* < 0.05), and NONate (*p* < 0.05; Figure 2h).

### 3.3. Coregulator Protein Levels

**Figure 3:**
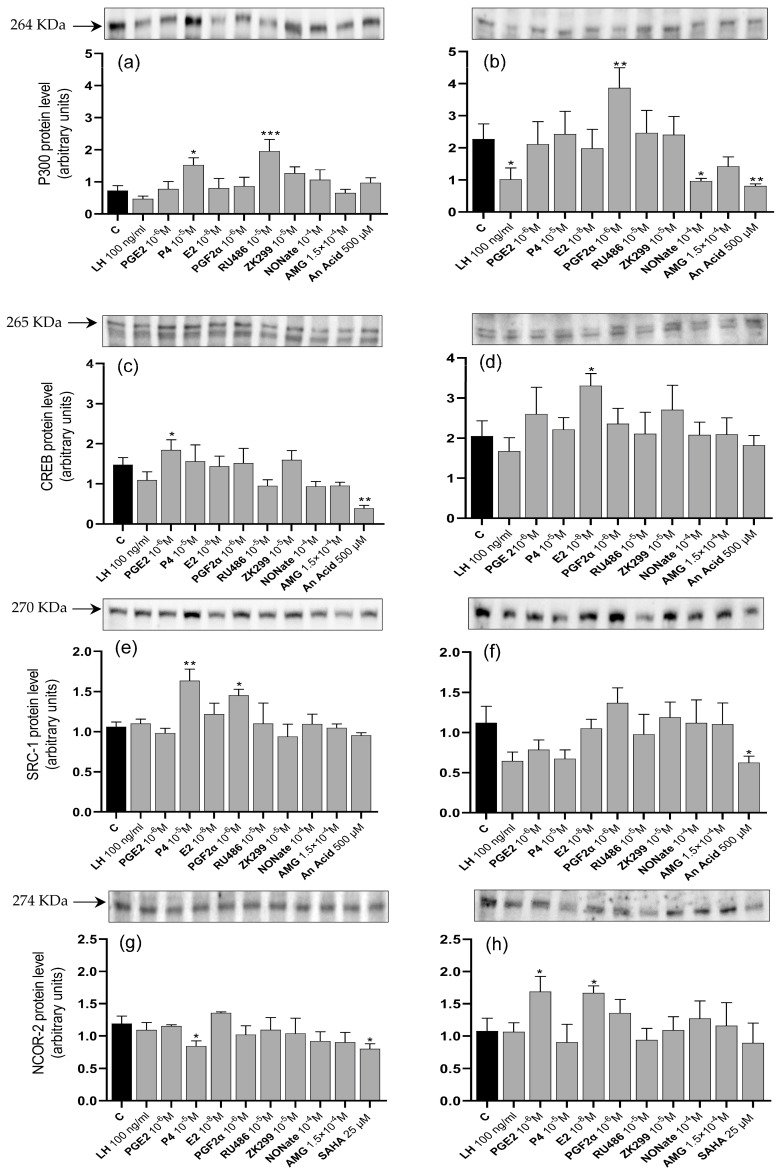
The mean (±SEM) of coactivators P300 (**a**,**b**), CREB (**c**,**d**), and SRC-1 (**e**,**f**) and corepressor NCOR-2 (**g**,**h**) protein levels in luteal cells from days 6 to 10 (**a**,**c**,**e**,**g**) and 17 to 20 (**b**,**d**,**f**,**h**) after 24 h treatment with LH (100 ng/mL); progesterone (P4) (10^−5^ M); prostaglandin E2 (PGE2) (10^−6^ M); estradiol (E2) (10^−8^ M); luteolytic factors prostaglandin F2α (PGF2α) (10^−6^ M), progesterone receptor inhibitors Mifepristone (RU486) (10^−5^ M) and Onapristone (ZK299) (10^−5^ M), nitric oxide donor (NONate) (10^−4^ M), aminoglutethimide (AMG 1.5 × 10^−4^ M); coregulator 500 μM AnAc; and corepressor 25 μM SAHA compared to untreated cells (C) (*n* = 4 in each group). The upper part shows a representative Western blot for each tested protein. Bars marked with asterisks are significantly different from the control (*, **, and *** represent *p* < 0.05, *p* < 0.01, and *p* < 0.001, respectively).

P300 protein levels in luteal cells from days 6 to 10 were increased by P4 (*p* < 0.05) and RU486 (*p* < 0.001; Figure 3a). In luteal cells from days 17 to 20, they were increased by PGF2α (*p* < 0.01) but decreased by LH (*p* < 0.05), NONate (*p* < 0.05), and an AnAc (*p* < 0.01) inhibitor (Figure 3b). In cells from days 6 to 10 of the estrous cycle, CREB protein levels were increased by PGE2 (*p* < 0.05) but decreased by an AnAc (*p* < 0.01) inhibitor (Figure 3c), while in luteal cells from days 17 to 20, they were increased by E2 (*p* < 0.05; Figure 3d). SRC-1 protein levels in luteal cells from days 6 to 10 were increased by P4 (*p* < 0.01) and PGF2α (*p* < 0.05; Figure 3e). In luteal cells from days 17 to 20, they were decreased by AnAc (*p* < 0.05; Figure 3f). NCOR-2 corepressor protein levels in luteal cells from days 6 to 10 were decreased by P4 (*p* < 0.05) and by a SAHA (*p* < 0.05) inhibitor (Figure 3g), whereas in luteal cells from days 17 to 20, they were increased by PGE2 (*p* < 0.05) and E2 (*p* < 0.05; Figure 3h).

### 3.4. HAT and HDAC Activities

**Figure 4:**
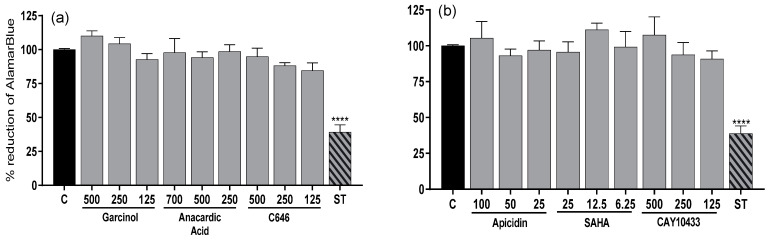
Percent reduction in Alamar Blue in luteal cells from days 6 to 10 of the estrous cycle incubated for 24 h with (**a**) HAT inhibitors garcinol (500, 250, and 125 μM), anacardic acid (AnAc) (750, 500, and 250 μM), and C646 (500, 250, and 125 μM), (**b**) HDAC inhibitors apicidin (100, 50, and 25 μM), SAHA (25, 12.5, and 6.25 μM), and CAY10433 (500, 250, and 125 μM), and staurosporine (ST, 500ng/mL) as a control reduction of Alamar Blue compared to untreated cells (C) (*n* = 4 in each group). Bars marked with asterisks are significantly different from the control (**** represents *p* < 0.0001).

Preliminary experiments showed that none of the used concentrations of the HAT (*p* > 0.05) (Figure 4a) and HDAC (*p* > 0.05) (Figure 4b) inhibitors lowered the Alamar Blue level relative to the control in luteal cells. Staurosporine as a control reduced the Alamar Blue for determining the inhibitors of HAT (*p* < 0.0001) (Figure 4a) and HDAC (*p* < 0.0001) (Figure 4b).

The inhibitors that lowered the activity of HAT were garcinol at a concentration of 250 μM (*p* < 0.05) and AnAc at a concentration of 500 μM (*p* < 0.0001; Figure S1a, Supplementary Data), whereas SAHA at a concentration of 25 μM reduced the HDAC activity in luteal cells (*p* < 0.0001; Figure S1b, Supplementary Data). AnAc at a concentration of 500 μM and SAHA at a concentration of 25 μM were the most effective HAT and HDAC inhibitors, respectively, and were used for further experiments.

None of the luteotropic or luteolytic factors altered the HAT (Figure S2a,b, Supplementary Data) or HDAC (Figure S2c,d, Supplementary Data) activity in luteal cells from days 6 to 10 (Figure S2a,c, Supplementary Data) and 17 to 20 (Figure S2b,d, Supplementary Data), whereas the HAT inhibitor reduced the activity in luteal cells from days 6 to 10 (*p* < 0.05) (Figure S6a, Supplementary Data) and days 17 to 20 (*p* < 0.01) (Figure S2b, Supplementary Data). In addition, the HDAC inhibitor reduced activity in luteal cells from days 6 to 10 (*p* < 0.05) (Figure S2c, Supplementary Data) and 17 to 20 (*p* < 0.05) (Figure S2d, Supplementary Data).

## 4. Discussion

This study has shown that luteotropic and luteolytic factors acting on a cow’s luteal cells can modulate the mRNA and protein levels of steroid hormone nuclear receptor coactivators (P300, CREB, and SRC-1) and corepressors (NCOR-2) on days 6–10 (mid-luteal) and 17–20 (luteal regression) of the estrous cycle without affecting the related HAT and HDAC activities, respectively.

The results show that on days 6–10, the luteotropic factors stimulated the expression of mRNA and proteins of most of the coactivators tested. These findings are in agreement with those of a previous report where we demonstrated that PGE2 increased nuclear PGR mRNA levels in bovine luteal cells on days 6–10 [41]. It has also been shown to be directly involved in using cholesterol from HDL for P4 synthesis [14]. P4 levels in CL tissue during the estrous cycle in cows revealed a correlation with the mRNA expression of all the coactivators presented in this publication [10]. This steroid hormone also stimulated the expression of key enzymes for steroidogenesis in bovine CLs: StAR, 3β-HSD, and cytochrome P450scc [18]. P4 alone did not affect the expression of its own nuclear receptor [42]. E2 contributes to PGR transcriptional activation through interactions with estrogen-responsive elements (EREs) in its promoter [17]. E2 was also observed to increase mRNA levels for the *PGRA* and *PGRB* isoforms in the bovine endometrium on days 6–10 [43]. Thus, the presented results, which show the action of luteotropic factors in luteal cells from the mid-luteal phase resulting in increased expression of mRNA and protein levels of tested coactivators, indicate that this mechanism may be an element supporting P4 action in cattle luteal cells. Therefore, in cow luteal cells, a change in mRNA expression of coactivators induced by luteotropic factors may be an important element that supports the action of nuclear steroid hormone receptors, including PGR.

The effect of PGR antagonists’ action has shown that only RU486 increased the mRNA or protein levels of coactivators. Previous studies have reported that the action of these compounds increased or decreased the expression of PGRA and PGRB isoforms in the bovine endometrium depending on their concentration [28]. Rothchild [44] reported that antagonists may reduce the effect of P4 when acting through the PGRA isoform, while their interactions with the PGRB isoform may cause the same effect as P4. This may also explain the increase in P4 levels after ZK299 treatment in our experiments. Therefore, we believe that the action of these inhibitors on coactivators may also be part of the described mechanism of regulation of nuclear PGRs. *PGRB* mRNAs are also expressed in cow CLs at significantly lower levels than *PGRA* mRNAs [2]. The active form of PGR binds to the gene promoter in the form of a dimer. PGRA and PGRB can bind as a homodimer A:A, a homodimer B:B, and a heterodimer A:B. Dimerization consequently modulates the transcriptional activities of PGRs and determines the diversity of physiological responses associated with P4 action. Therefore, despite its low mRNA expression, PGRB demonstrates an important function in regulating the CL.

The use of the luteolytic factors PGF2α and NONate in our study showed that on days 6–10, only PGF2α increased SRC-1 coactivator protein levels. Previous studies have shown that luteal PGF2α with oxytocin (OT) and P4 with noradrenaline (NA) play a luteotropic role in the early-to-mid bovine CL cycle. They create a positive feedback loop, stimulating P4 through a system of autocrine/paracrine interactions. Therefore, luteal cells were less sensitive to PGF2α, which could be a mechanism for preventing premature luteolysis. Considering these results, the increased expression of certain PGR coactivators and decreased expression of certain nuclear receptor corepressors on days 6–10 of the cycle caused by PGF2α may support its luteotropic action mechanism in the CL [45,46].

Contrary to coactivators, luteotropic and luteolytic factors reduced the expression of the NCOR2 corepressor in luteal cells from the mid-luteal phase. However, in cells from the luteal regression phase, NCOR2 expression increased under the influence of these factors. Thus far, it is known that corepressor binding to a nuclear receptor inhibits its activity, resulting in decreased expression of its target genes [47,48], whereas luteolysis is characterized by the cessation of the expression of key genes in CL functioning [49] and enhanced apoptosis [50]. Hence, the action of both luteotropic and luteolytic factors can help in providing an appropriate balance between coactivator and corepressor expression in the CL. Thus, it is possible that in the maturing CL, these factors tip the scale of this balance toward increasing coactivator mRNA expression. In contrast, in the luteal phase the balance is tipped towards increasing the mRNA expression of corepressors. Therefore, both groups of coregulators may be important and may be additional factors regulating the action of nuclear receptors for steroid hormones, including PGRs.

In luteal cells from days 6 to 10, most of the studied factors increased the coactivator’s expression, while on days 17–20, their effects were not so clear because these studied factors increased the expression of some of the coactivators and decreased others. To a large extent, increased mRNA levels of coactivators did not translate into the same increase in corresponding protein levels. In the absence of fertilization, the process of apoptosis is initiated, and changes in cell nuclei structure or increased endonuclease activity in the CL take place [51]. There is also a decline in the levels of P4 and mRNA and protein levels for both PGR isoforms [2]. In addition, the expression of genes important for CL function is ceased [52]. Therefore, the increased activity of these processes in luteal cells from days 17 to 20 of the cycle could translate into a lack of feedback and increases in the protein levels of the tested coactivators despite their increased expression could take place. Nevertheless, during this cycle interval, an increase in P300 protein levels was observed with PGF2α and CREB due to E2 action. The P300 and CREB coactivators bind to steroid hormone nuclear receptors, including the α (ERα) and β (ERβ) E2 receptors [53,54,55]. High ERβ receptor protein levels but low ERα levels were found in the regressive bovine CL [56]. E2 initiates the transcription of target genes by binding to ERα and inhibits their transcription by binding to ERβ [57]. Both PGF2α [56] and E2 [58] support CL luteolysis in the cycle’s final phase. Therefore, increasing P300 and CREB coactivator protein levels may support E2’s luteolytic activity by regulating ERβ receptor activity. Thus, it can be assumed that some of the coactivators whose expression is increased by the studied factors may be involved in the regulation of nuclear receptors participating in CL regression processes. On the other hand, for those coactivators whose action in these processes is reduced, expression is inhibited. Ultimately, this may have an effect in shifting the balance between processes that promote CL function and regression processes in favor of those that cause CL degradation.

Both mRNA expression and protein level results are very important in terms of the information provided. The level of mRNA directly reflects the action of the tested factors on changes in gene expression. Based on this, we can estimate how quickly and with what strength the cell responds to the factor. However, due to many regulations, mRNA levels do not always translate into protein levels. As the final product of translation, the protein level is closest to the phenotype of the individual. Thus, the protein level formed after the action of a given factor may more directly reflect its final effect on the cell [59].

The obtained results show that changes in mRNA expression of a given gene did not always translate into the same changes in the corresponding protein level. Potential reasons for this discontinuity include the many mechanisms regulating the protein translation process at the post-transcriptional and -translational levels [60]. One such mechanism may be gene expression inhibition by high levels of its encoded protein, as observed with the c-MYC protein in rats [61]. The process of mRNA expression and protein synthesis may be also shifted in time. Thus, the maximum synthesis of the protein occurs later than the highest expression of the gene, as was observed for OT synthesis [62]. These mechanisms are not yet sufficiently understood to directly calculate protein levels from mRNA levels.

The shift in protein synthesis relative to mRNA synthesis led us to adopt different times periods of stimulation of cells with factors for studying mRNA and protein expression. Our previous studies have shown that the maximum gene expression for enzymes of the steroidogenesis pathway was highest after 6 h of stimulation and declined thereafter [18]. This is also the time that we applied in the research presented in this publication. However, the maximum synthesis of the protein occurs later than the highest expression of the gene and requires more time. Therefore, we adopted 24 h from the start of stimulation to its end as the period in which all mRNA is transcribed into protein [18].

Initial experiments helped to identify AnAc as the optimal HAT inhibitor and SAHA as the optimal HDAC inhibitor. In further experiments, AnAc downregulated the expression of part of the tested coactivators, while SAHA downregulated the expression of the corepressor. High histone acetylation due to the stimulation of euchromatin formation increases the transcriptional activity of genes. Deacetylation is the opposing process that results in increased chromatin condensation and mRNA transcription inhibition [63]. AnAc and SAHA appropriately inhibit these processes. Maintaining cellular homeostasis requires balancing HAT and HDAC activity [64]. Disrupting this balance by inhibiting one of these processes may be associated with the over- or under-expression of genes critical for proper cell functioning [65]. Our HAT or HDAC activity experiments showed that AnAc and SAHA inhibitors, respectively, reduced activity, whereas none of the luteotropic and luteolytic factors changed HAT and HDAC activity in either cycle phase examined. This finding may indicate that the regulatory mechanism for nuclear receptor activity in the CL occurs through changes in mRNA and protein levels of individual coregulators, excluding changes in the activity of enzymes associated with them that regulate HAT and HDAC activities.

The analysis of changes in the level of P4 under the influence of the tested factors was considered as a verification of the usefulness of the cells for further stages of research and has already been used many times in our previous publications [14,18,41]. The response of the cells to LH treatment was observed for cells in both the studied estrous cycle stages. The P4 measurement after the addition of P4 detected both the hormone added to the medium and the hormone produced by the cells. Therefore, it cannot be definitively determined whether it stimulated its own synthesis. Therefore, these values are not shown on the graph. In luteal cells of both the studied phases, we did not show the PGF2α-induced inhibition of P4 synthesis. This process requires the presence of endothelin-1 (ET-1), which is secreted by endothelial cells [66]. In the case of the direct action of this prostaglandin on luteal cells (paracrine/autocrine action), the lack of ET-1 action may lead to an increase in P4 secretion as a result of PGF2α action [67,68].

It also has to be mentioned that isolated luteal cells were used as an in vitro research model through which all the intended scientific goals of this publication were achieved. However, one significant weakness of in vitro experiments is that they fail to reproduce the complexity of organ systems. Therefore, these results require further studies on an in vivo model to confirm the action of the studied factors at the tissue and whole-organism level.

To summarize the presented research results, we found that luteotropic and luteolytic factors influence changes in the mRNA and protein levels of coregulators and corepressors of nuclear receptors. Nevertheless, they did not affect the activities of HAT and HDAC related to coactivators and corepressors, respectively. Therefore, it is possible that these factors, through changes in the expression of nuclear receptor coactivators and corepressors independently of the effect on HAT and HDAC activity, may affect the action of the nuclear receptors, including PGRs, in the bovine CL.

## Figures and Tables

**Table 1:** Forward, reverse primer sequences used in Real-Time PCR. Every primer set was designed according to the accession number in the Nucleotide NCBI database.

Gene Name	Primers	GenBank Accession Number	Amplicon Length
*P300*	Forward: CCATGAGCAACATGAGTGCTAGT Reverse: CATTGTCACTCATCAGTGGGTTTT	XM_027540695.1	129
*CBP*	Forward: TGAAGTGAAGGTCGAAGCTAAAGA Reverse: GTACAGAGCTTCCAGGGTTGACAT	XM_024984694.1	147
*SRC-1*	Forward: CCCAGGCAGACGCTAAACAG Reverse: TCAAGATAGCTTGCCGATTTTG	XM_028514416.1	114
*NCOR-2*	Forward: AGCCCTCGAGGCAAAAGC Reverse: CATGCGGAGAGGCCTTGA	XM_024977670.1	177
*TBP*	Forward: CAGAGAGCTCCGGGATCGT Reverse: ACACCATCTTCCCAGAACTGAATAT	NM_001075742	194

## Data Availability

All the data presented in this study are included in the article.

## Supplementary Materials

The following supporting information can be downloaded at: https://www.mdpi.com/article/10.3390/ani13172784/s1, Figure S1. HAT (a) and HDAC (b) activities in luteal cells from days 6–10 of the estrous cycle incubated for 24 h. Figure S2. HAT (a,b) and HDAC (c,d) activities in luteal cells on days 6–10 (a,c) and 17–20 (b,d) of the estrous cycle incubated for 24 h with luteotropic factors.
