# Supplementary material for: Luteotropic and Luteolytic Factors Modulate the Expression of Nuclear Receptor Coregulators in Bovine Luteal Cells Independently of Histone Acetyltransferase and Histone Deacetylase Activities
Source: Animals (Basel). 2023 Aug 31;13(17):2784. doi: 10.3390/ani13172784 (PMC10486568; doi:10.3390/ani13172784)
Supplement: Supplementary file 1 [file animals-13-02784-s001.zip › animals-2349127-supplementary.docx]

b)

a)

**Figure S1.** HAT (**a**) and HDAC (**b**) activities in luteal cells from days 6–10 of the estrous cycle incubated for 24 h with (**a**) HAT inhibitors Garcinol (500, 250, and 125 μM), Anacardic Acid (AnAc) (750, 500, and 250 μM), and C646 (500, 250, and 125 μM), and (**b**) HDAC inhibitors Apicidin (100, 50, and 25 μM), SAHA (25, 12.5, and 6.25 μM), and CAY10433 (500, 250, and 125 μM) compared to untreated cells (C) (n=4 in each group). Bars marked with asterisks are significantly different from the control (*, and **** represent *p* < 0.05, and *p* < 0.0001, respectively).

a)

b)

d)

c)

**Figure S2.** HAT (**a**, **b**) and HDAC (**c**, **d**) activities in luteal cells on days 6–10 (**a**, **c**) and 17–20 (**b**, **d**) of the estrous cycle incubated for 24 h with luteotropic factors: LH (100 ng/mL); progesterone (P4) (10^−5^ M); prostaglandin E2 (PGE2) (10^−6^ M); estradiol (E2) (10^−8^ M) and the luteolytic factors: prostaglandin F2α (PGF2α) (10^−6^ M), progesterone receptor inhibitors: Mifepristone (RU486) (10^−5^ M) and Onapristone (ZK299) (10^−5^ M) as well as nitric oxide donor (NONate) (10^−4^ M); steroidogenesis inhibitor: aminoglutethimide (AMG 1.5 × 10^−4^ M) and respectively selected HAT and HDAC inhibitors 500 μM anacardic acid (AnAc), and 25 μM suberoylanilide hydroxamic acid (SAHA) compared to untreated cells (C) (*n* = 4 in each group). Bars marked with asterisks are significantly different from the control (* represent *p* < 0.05).
